# Hospital and Institutional News

**Published:** 1921-01-22

**Authors:** 


### HOSPITAL AND INSTITUTIONAL NEWS.

### THE PRINCE OF WALES AND THE COLLEGE OF SURGEONS.

H.R.H. the Prince of Wales has consented to be a guest at the Hunterian Festival Dinner on February 14, when he will receive the diploma of honorary fellowship of the Royal College of Surgeons to which he was elected on July 24, 1919.

### PAYING PATIENTS AND PROVIDENT PLANS.

In our issue of January 1, page 308, we referred briefly to the Brighton provident scheme for hospital benefits and additional medical services. The plan, organised by Dr. J. Gordon Dill, O.B.E., is the first of its kind that has appeared, and this fact, as well as its own merits, deserves that it should be studied by all hospital workers. We therefore have much pleasure in publishing full details on another page, in the hope that the information will be of service to those who are considering the advisability of producing provident schemes elsewhere.

### THE NATIONAL RELIEF FUND.

Apparently a good many hospitals are a little shy of mentioning gifts from the National Relief Fund, and very few of the grants have been announced. We understand, however, that the Bath Royal United Hospital has received £12,500, and this will help materially to reduce the deficit, which was £18,494 at the end of 1919. Our contemporary, *Truth*, thinks that the Fund has gone outside its original purpose in helping the hospitals, and says that such is the opinion of a hospital manager of London. Whether the hospital managed by the gentleman referred to received a grant or not, we are not informed. The Fund certainly was to relieve distress caused by the war, and there is little doubt that our hospitals are now relieving people whose conditions has been aggravated by the stress and privations of the war years, and that the distressful condition of the finances of hospitals has been largely caused by the war.

### A WELCOME GRANT.

Among the grants made to hospitals by the National Relief Fund is £20,000 to the Manchester Children's Hospital, Pendlebury. This admirable institution badly needed help, and is now enabled to pay off its indebtedness to the bank. In 1919 alone the deficit was £12,000, and one ward has been closed. It remains to be seen if the necessary income can be raised to prevent a further relapse. We hope it can, for the Children's Hospital at Pendlebury is deservedly famous, and should be a model in finance as it has been in administration.

### THE PATHOLOGY OF INFLUENZA.

In connection with our contributor's note upon the after-effects of influenza (p. 381), it is interesting to note that in the *American Journal of Experimental Medicine* account is given of an extensive series of researches carried out by Drs. Blake and Cecil in the laboratories of the Washington Army Medical School. A strain of the *B. influenza* was obtained directly from the pleura of an empyema case, the organism being, therefore, of a definitely pathogenic type. The virulence of the organism was then raised by successive passage through the bodies of a series of white mice and monkeys, and then, owing to the known fact that this organism rapidly loses its essential powers in sub-cultures outside the body, the respiratory passage and lungs of a second set of monkeys were inoculated with as little delay as possible. It is stated that in twelve animals the culture was simply applied to the mucous membrane of the nose and mouth, and of these five developed, apart from general prostration, acute suppuration in one or both mastoid antra, and two developed acute broncho-pneumonia yielding pure cultures of *B. influenza.* Another ten animals received intratracheal inoculation of the above cultures, and of these seven developed broncho-pneumonia and two showed tracheo-bronchitis. Post-mortem examination of these cases demonstrated lesions almost exactly paralleled by those seen in man during the acute outbreaks of 1918-19. Here we make but regrettably brief reference to a paper deserving close attention, for it appears to go far towards deciding that true influenza is actually due to the bacillus described by Pfeiffer.

### A DISCLAIMER.

We are glad to see that a disclaimer has appeared in the *B.M.J.* from Dr. Cranston Walker, whose note upon the value of adrenalin for resuscitation in accident cases, etc., was so absurdly misquoted in a section of the lay press last week and under the most sensational of headlines. It is little short of outrageous that serious facts and discussions should be thus taken from purely professional journals, written up by someone who has obviously little first-hand knowledge of the subject, and then published, names complete, without any preliminary permission from the authors concerned. It is bad enough that the public should be plied with ridiculously distorted reports of foreign medical work, or the sensational announcements notoriously originating with quacks in our own country, but that British scientific journals should also be made a happy hunting-ground is more than regrettable, and decidedly calls for editorial attention in the quarters concerned.

### SOME NOTABLE BEQUESTS.

There have been an unusually large number of bequests to hospitals and other charities announced lately, and some of quite important amounts. Lord Egerton of Tatlow has left, payable after the lifetime of his widow, £5,000 each to the Altrincham Provident Dispensary, the Hospital for Consumption, Brompton, and the General Lying-in Hospital, Lambeth. Other important bequests have been made by Mr. David Currie, a brother of the late Sir Donald Currie. These include £20,000 stock each to St. Thomas's, St. George's, St. Batholomew's,

Westminster, Middlesex, the London, Guy's, and Poplar Hospitals; £25,000 to Greenock Infirmary, and £5,000 each to Liverpool Royal Southern Hospital, Belfast Infirmary, King's College and St. Mary's Hospitals, and King Edward's Hospital Fund. All these legacies are in Canada, India, and New Zealand stock, and must be appraised at about half their face value, but, as Mr. Currie has expressed the wish that the money will not be expended on building operations, and that the income will be retained for the benefit of the patients, no doubt the stocks will be used for the desirable object of strengthening the respective endowment funds, and market value, for the moment, is not of great importance.

### HOSPITALS AND TELEPHONES.

Many hospital secretaries have received during the past week or so a notice to determine the existing telephone agreement. This no doubt will be followed by a fresh agreement for signature on the lines published recently in the newspapers. The change from a flat rate to a message rate will in many cases, apart from cost, require some check on the use of the telephone to ensure that all calls are on the business of the institution. An interesting move has been made by the Batley and District Hospital Board, who have decided to ask the British Hospitals' Association and the British Medical Association to use their influence to secure that telephone calls from hospitals shall be entirely free, in view of the serious expenditure that would be entailed in "calling" the hospital doctors. We should much like to hear our readers' views on the proposed new telephone rates and how they are likely to affect hospital expenditure and organisation.

### THE BRIGHTON BOXING TOURNAMENT.

We referred briefly in our issue of January 8 to the excellent work of Mr. H. J. Preston, of the Royal York Hotel, Brighton, on behalf of the hospitals and charities of that town, and, in connection with the very successful recent Boxing Tournament, stated, through a slip of the printer, that the Royal Sussex County Hospital would receive £300 instead of £3,006, as was the case. We understand that the Boxing Tournament last year was by way of an extra to Mr. Preston's annual effort at Christmas-time, which takes the form of a sale of dolls. As the last-named event produced £1,200, we surmise that there must have been a great many dolls or a great many generous buyers, or, as is likely, both. In any case Mr. Preston possesses the secret of success in these undertakings and the Brighton hospitals are very fortunate in having him as a friend.

### THE METROPOLITAN HOSPITAL'S NEW SECRETARY.

Mr. Herbert F. Rutherford, Secretary at the Beckett Hospital, Barnsley, has been appointed to succeed the late Mr. Dale as Secretary at the Metropolitan Hospital. There were upwards of 130 applicants for the post; the first short list consisted of eleven candidates, all of whom had hospital experience, and the second list of four. Mr. Rutherford, who is thirty years of age and was educated at the County School for Boys, Cambridge, commenced his hospital career at Addenbrooke's Hospital, Cambridge, where he received his training, under Mr. Richard J. Coles, Secretary Superintendent and now Secretary of King's College Hospital, London. Whilst at Addenbrooke's Mr. Rutherford had excellent opportunities of gaining a good knowledge of every branch of hospital administration. He joined the Army as a private in the early days of the war, and was subsequently given a commission and promoted to the rank of Captain. Upon demobilisation ten months ago he was appointed from a large number of applicants Secretary to the Beckett Hospital, Barnsley, as successor to Mr. Guy Dale, who had been appointed Secretary and House Governor at the Metropolitan Hospital. In this sphere of work Mr. Rutherford was most successful, and was instrumental in considerably increasing the annual income of the hospital. His many friends in the hospital world wish him every success in his new appointment, which he is to take up next month.

### THE ROYAL CHEST HOSPITAL.

As was expected, the meeting at the Mansion House on Monday last of the Governors of the Royal Hospital for Diseases of the Chest, City Road, approved of the amalgamation with the Great Northern Central Hospital. Mr. Alexander Wedderburn, the Vice-Chairman of the Chest Hospital, moved the formal resolution and explained the details of the proposed combination, which had previously been circulated and were dealt with in these columns last week. The general effect is that the distinctive character of the work will be maintained and the interests of both governors and staff will be duly observed. The present buildings in the City Road are to remain as the Royal Chest Section of the Great Northern Central Hospital until such time as building adjoining the Great Northern Central main building in Holloway Road becomes practicable, when the in-patient block and the nurses' home will be disposed of, but the out-patient department and *x*-ray department will continue as an outpost of the chest section of the Great Northern. The Marquis of Northampton, who is a governor of the Chest Hospital as well as Chairman of the Great Northern, assured the meeting that the special work which had for so long been carried on successfully would not suffer in any way.

### BIOLOGICAL STANDARDS.

In our recent reference to the work of the Medical Research Council we called attention to the value of standardised investigations upon the many biological preparations which modern usage is substituting for the older drugs. Earlier recommendations are being developed at the Institute in Hampstead, and a Special Committee on Biological Standards and the Methods of Biological Assay, consisting of Professor W. Bulloch, Dr. H. H. Dale, Professor Dreyer, Sir Walter Fletcher, and Professor C. J. Martin, has been appointed. Last April a departmental committee was appointed to advise the Ministry of Health on administrative and legislative measures that might be necessary in this connection, and its report is expected to appear shortly. Dr. Dale is to serve as Director of a Biological Standards Department at the National Institute, which should be an appropriate permanent home for an establishment the duties of which will be analogous in importance to the functions of the National Physical Laboratory.

### A COMING CONFERENCE.

The London County Council have decided to invite the Metropolitan Borough Councils to appoint representatives to attend a conference with members of the Public Health Committee and the Education Committee of the County of London to be held on Friday, February 25, for the purpose of considering (i) measures for the better control of measles in London, and (ii) measures for the more effective prevention of verminous conditions in London. It has been pointed out that the effective control of measles necessarily depends largely on the co-operation between the school organisation and the local health organisation, whilst a similar combination of effort is equally desirable for the prevention of verminous conditions. In both cases at present there is a lack of co-ordination, especially with regard to the cleansing of verminous persons. This duty was thrown on the London County Council to perform, but, as they had no cleansing stations in existence, whilst most of the borough councils had, they asked the latter to do the work. This they were agreeable to do, but considerable friction has occurred between the two authorities owing to the financial difficulties that have arisen, the councils maintaining that they were not adequately paid by the London County Council for the services they rendered the community. The conference will, no doubt, help to clear up these difficulties, and tend to a smoother working between all the authorities involved.

### ALLEGED HOSTILITY TO HOSPITALS IN MANCHESTER.

Mr. C. Swinglehurst, Chairman of the Manchester Hospital Saturday Fund, has drawn attention to a strange condition of affairs in Manchester. Declaring that such towns as Leicester, Newcastle, and Leeds collect three times more money than Manchester, he says: "A large number of employers and managers in our district refuse to allow collections or assist in any way." The reasons for this refusal would be worth knowing, and employers who take this hostile attitude without some explanation throw a very disagreeable imputation on themselves. We hope, therefore, that Mr. Swinglehurst will inform us why his fund meets with such scant respect, since those employers who refuse to have anything to do with it must have some reason for their attitude, and, this known, the reason can be discussed to the mutual advantage of the employers and of the fund. After all, as he pointed out in a recent letter, if the voluntary hospitals went upon the rates, it would be the employers who would have to provide most of the money. It would seem, then, that those who brush aside the claims of the Saturday Fund must be very stupid or very hostile, and either frame of mind can, perhaps, be improved if its assumptions are examined carefully.

### PAYING PATIENTS IN POOR-LAW INFIRMARIES.

In Manchester also the Guardians are trying to relieve the waiting-lists at the voluntary hospitals by offering for moderate fees institutional treatment. They hope to provide limited accommodation for paying patients at their Crumpsall, Nell Lane, and Withington Hospitals. The charge will be three guineas a week, and will cover the cost of maintenance and treatment. The patients will be treated as they would be in a private institution, and the Guardians' hospitals are well equipped and possess a visiting staff of physicians and surgeons. It is interesting to remark that the Guardians declare that their action is not due to a number of vacant beds. They say that their patients are almost as numerous as they were before the war, but that they have a limited amount of accommodation which can be used for private patients in connection with their existing staffs. At all events, their proposal will be welcomed, and, if it can be made a success, it will encourage similar arrangements elsewhere.

### INFIRMARY REFORMER'S DEATH.

Dr. J. Breward Neal, who for over thirty years was medical superintendent of St. John's Hill Infirmary, Wandsworth, passed away at the age of seventy-four years. He retired from the Guardians' service in 1913, but pluckily returned during the war, and did much excellent work for wounded soldiers. It was only a few days before his death, which occurred suddenly, that Dr. Neal paid a visit to the infirmary. He came to Wandsworth when the attacks were severe on the old methods of treating the sick poor, and he did much to remove many of the odious associations then connected with Poor-law infirmaries. It was mainly through his efforts that Wandsworth Infirmary has been raised to the position of one of the foremost institutions in the Metropolis.

### SENTIMENT AND SUSPICION AT CARDIFF.

The new chairman of the board of management of King Edward VII. Hospital, Cardiff, Sir W. H. Diamond, took an early opportunity to state the policy which he favours. He declared at the first meeting over which he presided that unfortunately the people of Cardiff have regarded the hospital too much from a sentimental point of view. It should, he added, be recommended as practical and necessary. At present he complained that there is "a blighting atmosphere of suspicion" which makes it difficult to carry on the hospital. The internal rules require to be brought up to date. Many people are brought to the hospital who should not be there, and these help to swell the waiting-list. Their first business must be to consolidate their income. We are not clear whether Sir William thought that the blight to which he referred was the result of the sentiment that he deplored, or whether he thought that people would give more if less appeals were addressed to their feelings and more to their public spirit. Surely it would be a mistake to neglect either argument. We shall await with interest Sir William's case when next he presents the hospital's claims to his fellow-citizens.

### A NEW HOME IN SOUTHGATE PARK.

In February the Great Northern Central Hospital will take over as a hospital of recovery the former auxiliary war hospital, known as "Grovelands," in Southgate Park, which the Southgate Voluntary Aid Association has placed at its disposal. Grovelands will provide at first thirty-five beds, and thus take the place of the temporary home at Summerlee, East Finchley, which is being closed. If sufficient financial support is obtainable, further beds can be added, for the house, which was designed by Nash, can accommodate seventy patients. The Southgate V.A.D. has added largely to the size of the grounds, which now consist of thirteen acres. Full particulars were lately given in *Progress*, a paper run by the Southgate V.A.D.

### WHAT A LINEN GUILD CAN DO.

In many things Swansea Hospital is fortunate. The works employees who promised to give £20,000 last year, as a matter of fact have given nearer £22,000. This, as Mr. Roger Beck says, is "doing magnificently." Many employers also doubled their subscriptions. Besides this the institution has the advantage of an admirable Linen Guild. The assistance of the guild is valued at £1,000 a year, and till recently it supplied the hospital with its bedding and paid for the uniforms of the staff. Now, however, because the increased price of materials and the more numerous staff, the Guild feels itself unable to shoulder the latter responsibility. A comparison of the value of linen guilds to different institutions would be interesting. How many can boast of over £1,000 a year?

### SHOULD LOCK HOSPITALS CHANGE THEIR NAMES?

It has been decided to rechristen the Manchester and Salford Lock Hospital, and to change its name to St. Luke's Hospital, Duke Street, Manchester. The medical officers report that the old name of the hospital, which indicated so unmistakably the nature of the diseases treated, deterred patients from attending. If this is so the decision cannot be criticised, though one may regret the flaccidity of an age which can speak only in euphemisms.

### THE FIRST TRANSFERRED POOR-LAW HOSPITAL.

Writing to the Bradford Corporation in regard to St. Luke's Hospital, Dr. Addison expresses appreciation of the ready way in which his desire for consultation with the local medical profession had been met, and that a practical means of providing for such consultation had been so readily devised. He assumes that steps will now be taken by the Health Committee to obtain formally the views of the Local Medical Advisory Committee on the St. Luke's Hospital scheme so far as it raises issues directly or indirectly affecting, or including, the medical profession. These issues are of a very great importance, not only in Bradford but in relation to the future provision of health services for the county as a whole, and Dr. Addison is persuaded that any delay which may follow from formal consultation with the local committee will not be lost time. He hopes at an early date to have the privilege of approving a scheme for the first transferred Poor-law hospital, which will represent agreement between the local authority and the local medical profession speaking through its authorised representatives.

### REJUVENATED BAD FOOD.

Southwark Borough Council is to be commended for its decision to invite the other Metropolitan borough councils to assist in stopping a practice which unfortunately in some districts has become a danger to the community. Attention had been called to the measures taken in London for preventing condemned food from being again offered for sale for human consumption. Attempts are sometimes made to purchase quantities of condemned food, ostensibly for pig feeding, but actually with the intention of placing the same food, by a process of circumrotation, before the public as a sound commodity. The measures adopted in Southwark are some safeguard against such practices, but it is doubtful whether similar precautions are taken in all London boroughs. A recent case showed that the condemned food was taken from the one borough and sold in another.

### ABERAYRON COTTAGE HOSPITAL OPENED.

Miss Lewes, O.B.E., of Tyglyn Aeron, has formally opened the new cottage hospital at Aberayron. The hospital possesses a theatre and an *x*-ray apparatus, which was chosen with the advice of Dr. Garfield Evans, of Cardiff, who is regarded as the chief specialist in this subject in Wales. We are glad to note that Mr. Felix, who is Chairman of the Board of Guardians, has promised a subscription of £50, provided that other boards will contribute similarly.

### THIS WEEK'S DRUG MARKET.

There is still no improvement in business, and the tendency for the weak holders to unload their stocks at "bargain" prices is rather more pronounced. Even offers below general current rates do not, however, seem to lead to anything remarkable in the way of business, buyers seeming to be under the impression that if they wait a little longer values will still be lower. They find support in this belief in the fact that drugs in considerable variety are coming over from the Continent in greater quantity and at rates lower than those recently ruling. So far as synthetic products are concerned the demand continues extremely dull, notwithstanding the reduced prices. Cocaine is again cheaper. Quinine is offered at substantially below makers' prices. Permanganate of potash is plentiful and cheaper. Potassium bromide is offered at remarkably low prices.

